# *Solanum nigrum* L.-derived nanovesicles as novel nanotherapeutics suppressing prostate cancer progression via senescence-based antitumor activity

**DOI:** 10.1186/s40643-026-01055-y

**Published:** 2026-04-27

**Authors:** Miao Gan, Yufei Feng, Luhua Xu, Zetao Chen, Fanjia Zhong, Na Liu, Jiamei Huang, Huizhen Zhang, Jin Tu, Chao Yang, Rongfeng Yang, Fengxia Lin

**Affiliations:** 1 Department of Cardiovascular, Shenzhen Bao’an Traditional Chinese Medicine Hospital, Guangzhou University of Chinese Medicine, Shenzhen, 518100 China; 2 Seventh Clinical Medical College, Guangzhou University of Chinese Medicine, Shenzhen, 518100 China; 3 Division of Cardiovascular Intensive Care (CICU), Cardiac and Vascular Center, The University of Hong Kong-Shenzhen Hospital, Shenzhen, 518053 China; 4 Department of Andrology, The Central Hospital of Enshi Tujia and Miao Autonomous Prefecture, Enshi, 445000 Hubei China; 5 School of Traditional Chinese Medicine, Guangdong Pharmaceutical University, Guangzhou, 510006 China; 6 Department of Urology, Shenzhen Clinical College of Integrated Chinese and Western Medicine, Guangzhou University of Chinese Medicine, Shenzhen, 518104 China

**Keywords:** Plant-derived nanovesicles, Senescence, Prostate cancer, P53, Herbal medicine

## Abstract

Current therapies for prostate cancer are limited by toxicity and acquired resistance, motivating development of biocompatible nanotherapeutics. Here, *Solanum nigrum* L.-derived nanovesicles (SDNVs) were isolated and characterized, showing a mean diameter of 93.4 ± 0.2 nm and a particle concentration of (4.2 ± 0.4) × 10^11^/mL. SDNV uptake, antitumor activity, and safety were assessed in PC-3 and RWPE-1 cells, while efficacy and biodistribution were further evaluated in a subcutaneous PC-3 xenograft mouse model following oral administration. SDNVs were readily internalized by PC-3 cells and suppressed viability, proliferation, and migration while inducing apoptosis. Senescence and cytotoxicity were not observed in RWPE-1 cells, indicating tumor-selective activity. The underlying mechanism was further investigated by transcriptome sequencing, followed by quantitative PCR, Western blotting, and pharmacologic rescue with the p53 inhibitor pifithrin-α (PFT-α). Transcriptomic analysis revealed 5058 differentially expressed genes, and further indicated activation of the p53/p21 axis and promoted cellular senescence in PC-3 cells after SDNV treatment. In vivo, oral SDNVs significantly reduced xenograft growth and showed no evident histopathologic toxicity in major organs. Collectively, SDNVs represent a plant-derived, biocompatible nanotherapeutic platform that selectively restrains prostate cancer growth via p53/p21-mediated senescence, supporting further development of senescence-targeting SDNV-based interventions.

**Graphical abstract:**

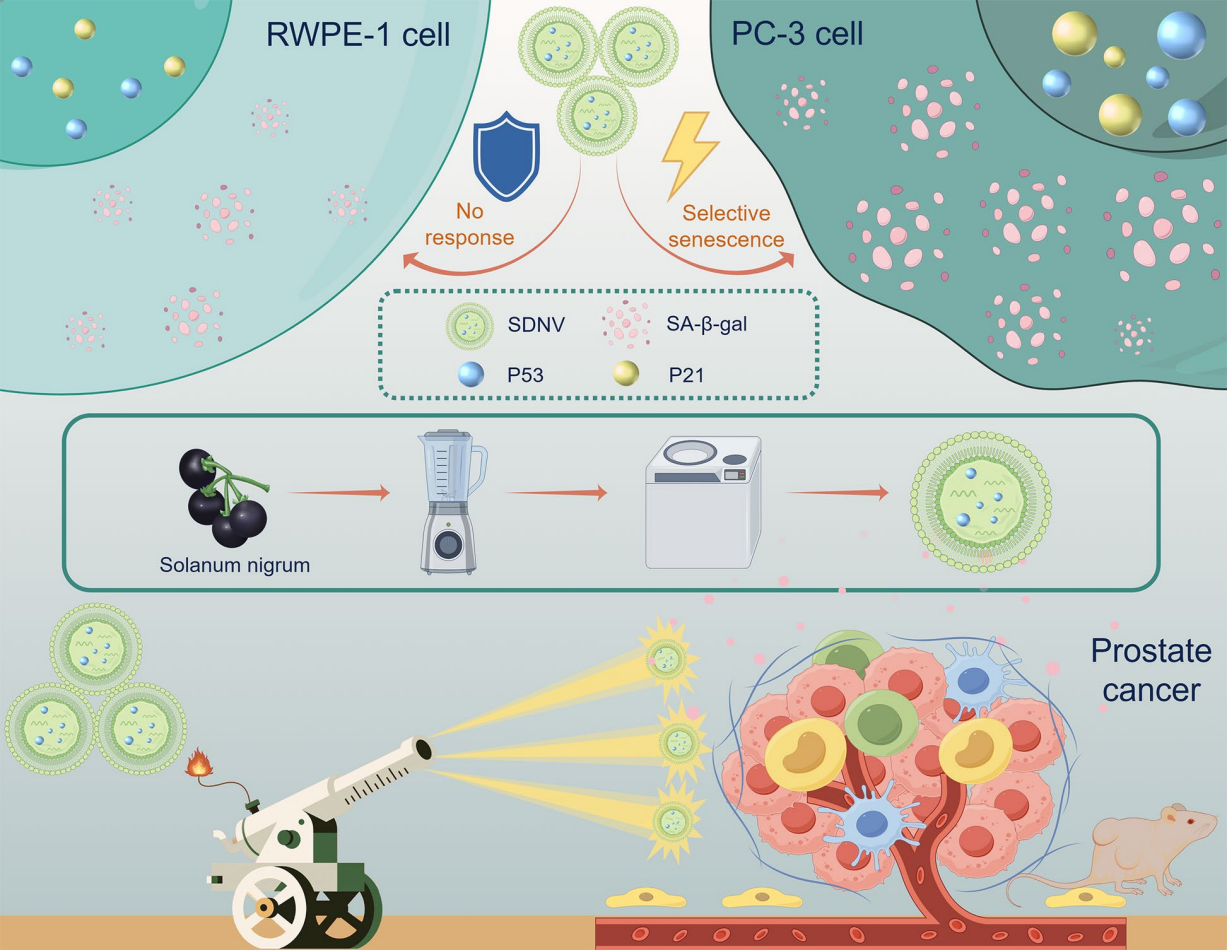

**Supplementary Information:**

The online version contains supplementary material available at 10.1186/s40643-026-01055-y.

## Introduction

As a common urologic malignancy in aging males, prostate cancer (PCa) continues to impose a substantial global health burden (Rebello et al. 2021). In 2022, PCa was responsible for approximately 1.47 million new incident cases and nearly 0.40 million deaths, making it a major contributor to cancer-related morbidity and mortality (Nierengarten 2024). Although several therapeutic strategies, including chemotherapy, radiation therapy, hormone therapy, surgery, and cryotherapy, are currently used for treating PCa, these techniques face limitations due to major side effects, drug resistance, poor tumor selectivity, and high costs (Livak and Schmittgen 2001; Termini et al. 2020; Mashele 2022). Therefore, developing novel therapeutic options with higher efficacy, fewer adverse effects, and improved selectivity is urgently needed. Cellular senescence is a well-established antitumor program characterized by irreversible growth arrest, increased lysosomal activity, DNA damage accumulation, and secretion of numerous bioactive factors. The process is mainly regulated by signaling pathways including p53/p21 and p16INK4a/Rb (Gorgoulis et al. 2019). Senescent cells also exhibit hallmark features including elevated lysosomal content, distinct morphological changes, epigenetic alterations such as senescence-associated heterochromatic foci, and nuclear membrane remodeling (DeLuca and Saleh 2023). In cancer, senescence plays a dual role—suppressing tumor cells while potentially promoting progression when present in the tumor microenvironment (Schosserer et al. 2017). In PCa, p53-mediated senescence is critical for restraining tumor growth and overcoming therapy resistance. PTEN loss in prostate epithelium triggers a p53-driven senescence mechanism, inhibiting malignant transformation (Chen et al. 2005). In Pten-deficient mice, early lesions such as prostatic intraepithelial neoplasia exhibit stabilized p53 and growth arrest with senescence features (Yanushko et al. 2025). These findings support senescence induction via p53 as a promising therapeutic strategy in PCa.

Recently, plant-derived nanovesicles (PDNVs) have attracted increasing interest as natural nanovesicles with therapeutic potential (Yi et al. 2025). Owing to their favorable biocompatibility, minimal immune response, and capability to cross biological barriers, they are considered promising candidates for therapeutic development. Emerging studies have demonstrated that PDNVs exhibit beneficial effects in various disease contexts, including inflammatory disorders, cardiovascular disease, and cancer (Kim et al. 2022; Yang et al. 2024; Lin et al. 2025). PDNVs have demonstrated promise in suppressing tumor progression through multiple mechanisms, such as inducing apoptosis, modulating immune responses, and disrupting angiogenesis (Chu et al. 2024).

*Solanum nigrum* L. is a traditional medicinal herb containing diverse bioactive constituents, including alkaloids, polyphenols, polysaccharides, and steroidal glycoalkaloids. Research indicates that its anticancer properties primarily involve suppressing tumor growth and triggering apoptosis (Yang et al. 2016; Lan et al. 2023). However, the potential of *Solanum nigrum* L.-derived nanovesicles (SDNVs) in cancer therapy, particularly for PCa, remains largely unexplored. Here, we propose that SDNVs may selectively suppress PCa progression by inducing tumor cell senescence via the p53/p21 pathway, without affecting normal cells. This work presents the first systematic investigation of SDNVs in PCa and aims to provide a novel therapeutic strategy supported by mechanistic evidence.

## Materials and methods

### Isolation and characterization of SDNVs

Dried *Solanum nigrum* L. fruits were homogenized using a blender with an appropriate volume of ultrapure water for 3 min to obtain the crude extract. SDNVs were isolated by differential centrifugation at 300 × g, 2000 × g, and 10,000 × g for 30 min, followed by ultracentrifugation at 135,000 × g for 70 min (Rebello et al. 2021). The pellet was resuspended in PBS and further purified by discontinuous sucrose gradient ultracentrifugation using 15%, 30%, 45%, and 60% sucrose. The vesicle-enriched fraction at the 15–30% interface was collected, centrifuged at 150,000 × g for 2 h, resuspended in Tris–HCl buffer, and then passed through a 0.22 μm polyethersulfone (PES) membrane filter with low-protein-binding (Merck Millipore, Cork, Ireland) to obtain a clarified, sterile SDNV suspension (Lin et al. 2025).

For morphological assessment, SDNVs were negatively stained and examined by transmission electron microscopy (TEM) with a Tecnai G2 Spirit Twin (FEI, Eindhoven, The Netherlands) equipped with a Gatan 832.10W camera (Gatan, Pleasanton, USA). Nanoparticle tracking analysis (NTA) using a NanoSight NS300 system (Malvern Panalytical, Malvern, UK) was employed to measure the size and concentration of particle (Kim et al. 2024). Total protein content of SDNVs was measured with a BCA assay kit (Biosharp, Hefei, China). Protein identity and purity were further evaluated using Coomassie brilliant blue staining. The Agilent 2100 Bioanalyzer was used to quantify the total RNA content in SDNVs (Agilent, Santa Clara, USA).

### Metabolomics analysis

Metabolomic profiling of SDNVs was utilized ultra-high-performance liquid chromatography-tandem mass spectrometry (UHPLC-MS/MS) (Lin et al. 2025). Briefly, SDNV samples, water decoction of *Solanum nigrum* L. (SWD), and the reference standards solasonine were treated with methanol for protein precipitation and then centrifuged to collect the supernatants. These samples were subsequently assessed on a Vanquish UHPLC system coupled to a Q Exactive HF-X (Thermo, Bremen, Germany) (Want et al. 2013).

### Raman spectroscopy

SDNV samples suspended in PBS were placed on a glass slide for analysis using a LabRAM HR Evolution Raman microscope (Horiba, Kyoto, Japan). After initial optical focusing, a second laser focusing step was performed to optimize photon collection. Spectra in the 50–4000 cm^−1^ range were obtained with 785 nm excitation laser in single-point mode. Laser power, grating settings and integration time were adjusted based on preliminary test scans to obtain adequate signal intensity without sample damage. For each sample, spectra were collected from at least three randomly selected points and then exported for baseline correction, normalization and peak assignment.

### Lipidomic analysis of SDNVs

Lipidomic profiling of SDNVs was performed by LC–MS/MS. Equal amounts of purified SDNVs (normalized by protein content) were extracted utilizing a liquid–liquid extraction method based on methyl tert-butyl ether (MTBE) in the presence of a SPLASH™ internal standard mixture (Matyash et al. 2008). Lipids were analyzed by UHPLC-MS/MS. Raw data were analyzed with LipidSearch and XCMS. Identified lipid species were grouped into major classes (e.g. PC, PE/PEt, PI, PS, PG, LPC, DGDG, MGDG, SQDG, SM, Cer/HexCer, DG, TG), and class-level abundances were calculated by summing all molecular species within each class and expressing the result as percentage of total identified lipids.

### Cell culture, fluorescent labeling, and uptake of SDNVs

The RWPE-1 normal prostate epithelial cells and the PC-3 prostate cancer cells and were used from ICELL (Shanghai, China). RWPE-1 cells were maintained in Keratinocyte Serum-Free Medium with bovine pituitary extract and epidermal growth factor (Gibco, Grand Island, USA). PC-3 cells were maintained in RPMI-1640 medium 10% fetal bovine serum with 1% penicillin–streptomycin (Gibco, Grand Island, USA) (Xie et al. 2021). All cells were incubated at 37 °C in an incubator with 5% CO_2_.

For fluorescent labeling, SDNVs (equivalent to 100 mg total protein, suspended in PBS) were incubated with DiR or DiI (Macklin, Shanghai, China), or DiO (Zeta Life, USA) at 37 °C for 30 min. After labeling, SDNVs were rinsed and resuspended in sterile PBS (Takov et al. 2017).

To evaluate cellular uptake, PC-3 cells grown on glass-bottom dishes were exposed to DiI-labeled SDNVs (1 × 10⁹ particles/mL) for 6 h. After removal of free vesicles by PBS rinsing, the cells were paraformaldehyde-fixed and visualized on LSM800 confocal microscope (Zeiss, Oberkochen, Germany). To assess uptake efficiency, PC-3 cells were treated with DiO-labeled SDNVs for 0, 1, 3, 6, 12, and 24 h and analyzed by flow cytometry (BD FACSCanto II) using the FITC channel (Escrevente et al. 2011). All experiments conducted in triplicate.

### Cell viability, proliferation and apoptosis assays

For cell viability analysis, PC-3 cells were treated with SDNVs at 0, 12, 18, 24, 30, 36, 48 μg/mL for 24 h. The CCK-8 kit (Abbkine, Wuhan, China) was introduced, and absorbance was read at 450 nm using a BioTek microplate reader (Winooski, USA). The results were standardized against untreated controls (Chamchoy et al. 2019). Experiment was performed three independent times.

PC-3 cells exposed to 36 μg/mL of SDNVs for 24 h, followed by assessment of cell proliferation and apoptosis. Post-treatment, cell proliferation was assessed using an EdU assay kit (Beyotime, Shanghai, China; C0078S). Nuclei were counterstained with Hoechst 33342. Fluorescence microscopy was used to visualize EdU-positive cells and total nuclei, which appeared as green and blue signals. Proliferation was quantified as the proportion of EdU-positive cells among total nuclei (Subramanian et al. 2005). For apoptosis, cells were evaluated by Annexin V/ PI apoptosis detection kit (Abbkine, Georgia, USA; KTA0002) and quantified on a FACSCanto II (BD Biosciences, San Jose, USA). The percentage of apoptotic cells (early + late) was quantified (Vermes et al. 1995). All experiments were conducted in triplicate.

### Cell migration assay

Cells suspended in 100 μL serum-free medium were seeded into the upper chamber of Transwell inserts (8.0 μm; Labselect, Hefei, China), while 400 μL complete medium containing 10% FBS was added to the lower chamber. After 12 h, non-migrated cells were gently removed, and migrated cells were stained with crystal violet after fixation. Migrated cells were counted in five random fields under an inverted microscope (Olympus, Tokyo, Japan) (BOYDEN 1962). Experiment was performed three independent times.

### β-Galactosidase staining assay

Cellular senescence in PC-3 cells was assessed by Senescence β-Galactosidase Staining Kit (Beyotime, Shanghai, China) after treatment with SDNVs (50 μg/mL) for 24 h. SA-β-gal-positive cells were quantified in five randomly fields under light microscope. Experiment was performed three independent times.

### Transcriptomic analysis of SDNV-treated cells

RNA libraries were constructed and then sequenced on a NovaSeq 6000 platform. After preprocessing with fastp, reads were aligned to the GRCh38 human reference genome with HISAT2 (Kim et al. 2019). Analysis was conducted with DESeq2, and genes with |log2FC|≥ 1 and adjusted p < 0.05 were considered differentially expressed (Love et al. 2014). KEGG pathway enrichment was analyzed with clusterProfiler (v4.6.2) (Yu et al. 2012).

### Xenograft model and tumor evaluation

Male BALB/c nude mice (8-week) were used for xenograft establishment. After one week of acclimatization, xenografts were established by subcutaneously injection of 1 × 10⁷ PC-3-Red-Fluc cells into the right flank of each mouse. Upon tumors reaching approximately 20 mm^3^, mice were assigned to four groups: a control group receiving PBS and three SDNV-treated groups with does of 0.18, 0.54, and 1.62 μg/μL, each administered 100 μL per mouse (n = 6). Oral administration was performed once daily for 28 consecutive days. Tumor size was recorded every 3 days with caliper, and volumes were derived using the formula V = 0.5 × length × width^2^ (Tomayko and Reynolds 1989). After the final treatment, tumors were harvested for weight measurement.

Hematoxylin and eosin (H&E) staining was performed to assess the changes in tissue architecture, necrosis, and cellular morphology. Apoptotic was assessed by TUNEL assay. Cell proliferation was evaluated via immunohistochemical staining for Ki-67. Ki-67 immunostaining was performed on paraffin-embedded tumor sections after citrate-based antigen retrieval (pH 6.0), and then incubated at 4 °C for 12 h with anti-Ki-67 antibody (Servicebio; 1:500). Staining was developed with HRP-conjugated secondary antibody and DAB, with hematoxylin used for nuclear counterstaining. Positive Ki-67 staining was visualized as brown nuclear staining and quantified by counting Ki-67-positive nuclei in five random fields.

### Biodistribution and biosafety evaluation of orally delivered SDNVs

The biodistribution of SDNVs was evaluated as previously described (Yang et al. 2024). When tumors reached approximately 20 mm^3^, DiR-labeled SDNVs were given to tumor-bearing mice by oral gavage at 1 × 10^11^ particles/kg once daily for 7 days. Six hours after the last dose, the heart, liver, spleen, lungs, kidneys, and tumor were harvested for fluorescence imaging on a Tanon ABLX3 system (Tanon, Shanghai, China). The intensity of DiR signal in different organs was used to evaluate the biodistribution pattern of orally administered SDNVs.

For biosafety assessment, major organs were sampled at the study endpoint, immersed in 4% paraformaldehyde for 24 h. Then, paraffin sections were prepared and examined by H&E staining. Potential tissue toxicity was evaluated by microscopic examination of stained sections. Body weight was monitored every 3 days throughout the 4-week treatment period. Blood was collected by cardiac puncture immediately after euthanasia. Serum was collected after centrifugation, and serum biochemistry was assessed for liver and kidney function markers, including ALT, AST, BUN and CRE.

### Quantitative real-time PCR

Total RNA was isolated from PC-3 cells and cDNA was generated with a reverse transcription kit (Vazyme, Nanjing, China). Quantitative real-time PCR (qPCR) was conducted with the SYBR Green qPCR premix (Vazyme, Nanjing, China) on a LightCycler 480 instrument (Roche, Basel, Switzerland). The expression levels of TP53, P21, and P16 were analyzed. Relative transcript levels were quantified by the 2^−ΔΔCt^ method (Livak and Schmittgen 2001). Primer sequences can be found in Additional file 1: Table S1.

### Western blot

Total protein was extracted with RIPA lysis buffer (Yeasen, Shanghai, China) and analyzed by western blotting following SDS-PAGE and transfer. After blocking, blots were incubated at 4 °C for 12 h with primary antibodies: anti-P53 (ZENBIO, Chengdu, China; 345567, 1:1000), anti-P21 (ZENBIO, Chengdu, China; 381102, 1:1000), and anti-GAPDH (Proteintech, Rosemont, USA; 60004-1-Ig, 1:5000). After incubation with HRP-conjugated secondary antibodies (EARTH, San Francisco, USA; E030110-01, 1:10,000) for 1 h, signals were detected by enhanced chemiluminescence (ECL) detection kit (Biosharp, Hefei, China), and images were captured using a Tanon 5200 imaging system. Target protein levels were measured using ImageJ denstitometry and normalized against GAPDH.

### Statistical analysis

Data are shown as mean ± SD. Data analysis was conducted using unpaired Student’s *t* test or one-way ANOVA, with statistical significance set at *p* < 0.05.

## Results

### Physicochemical features of SDNVs

**Fig. 1:**
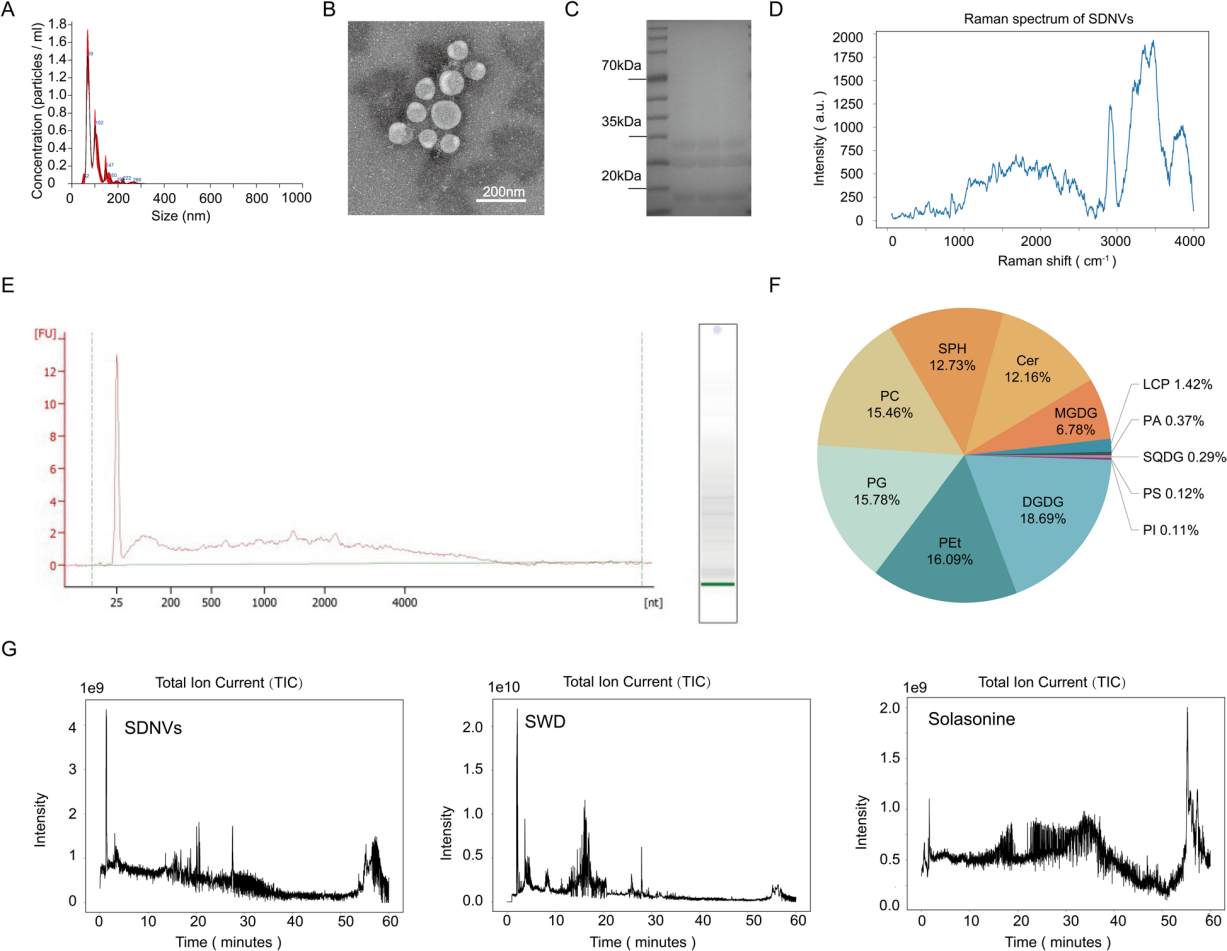
Identification and characterization of SDNVs. **A** Nanoparticle tracking analysis (NTA) illustrating the particle size distribution of SDNVs. **B** Representative transmission electron microscopy (TEM) image displaying typical morphology of SDNVs (scale bar, 200 nm). **C** SDS-PAGE and Coomassie brilliant blue staining revealing protein profiles of SDNVs. **D** Raman spectrum of SDNVs showing characteristic bands of lipid acyl chains, phosphate ester groups and protein/polysaccharide-associated hydroxyl and amino groups. **E** Agilent 2100 Bioanalyzer electropherogram confirming the presence of small RNAs within SDNVs. **F** LC–MS/MS-based lipidomic profiling of SDNVs at the class level, presented as the relative abundance of major membrane-associated lipid classes. **G** TIC chromatograms of SDNVs, Solanum nigrum decoction and solasonine, illustrating the distinct metabolite fingerprint of SDNVs

SDNVs were obtained from *Solanum nigrum* L. fruits using an optimized procedure that integrated differential ultracentrifugation with sucrose density gradient centrifugation. Under TEM, these vesicles exhibited typical round or oval morphology with bilayer membranes and diameters between 50 and 120 nm (Fig. 1A). NTA indicated a relatively uniform particle population, with vesicles averaging 93.4 ± 0.2 nm in diameter and reaching a concentration of (4.2 ± 0.4) × 10^11^ particles/mL (Fig. 1B). Coomassie Brilliant Blue staining demonstrated distinct protein bands primarily distributed in the 15–35 kDa range (Fig. 1C), indicating that SDNVs carry diverse endogenous proteins. Raman spectroscopy further characterized the chemical composition of SDNVs (Fig. 1D). Absorption peaks located at 3475–3361 cm^−1^ were assigned to hydrogen-bonded O–H and N–H stretching, consistent with hydroxyl- and amino-containing constituents associated with proteins and polysaccharides. A prominent band at 2913 cm^−1^ together with C–H bending modes at 1456–1388 cm^−1^ indicated saturated -CH_3_/-CH_2_ alkyl chains, consistent with lipid acyl chains, and a band at 845 cm^−1^ corresponded to P–O stretching in phosphate ester groups. Semi-quantitative analysis showed that free hydroxyl, protein/polysaccharide-associated hydroxyl, amino, saturated alkyl and phosphate groups accounted for 30%, 20%, 9%, 18% and 2% of the integrated signal, respectively. Characteristic Raman peaks of graphitized carbon were absent at approximately 1350–1580 cm^−1^, further excluding contamination from carbonaceous particles. Together with the TEM data, these Raman features support that SDNVs are organic nanovesicles enriched in membrane lipids and surface protein/polysaccharide moieties. Agilent Bioanalyzer analysis confirmed the presence of small RNAs in SDNVs (Fig. 1E), demonstrating that the vesicles also carry nucleic acid cargo. LC–MS/MS-based lipidomic profiling further defined the membrane lipid composition at the class level (Fig. 1F). Multiple phospholipid, glycolipid, and sphingolipid classes were detected, supporting the presence of a lipid bilayer-based vesicular membrane. In addition, LC–MS metabolomic profiling showed that the total ion current (TIC) fingerprint of SDNVs was clearly distinct from both the water decoction of S. nigrum and purified solanine (Fig. 1G), suggesting that SDNVs represent a unique bioactive fraction of the plant that is not captured by traditional decoction-based extraction.

### Biodistribution of orally administered SDNVs

**Fig. 2:**
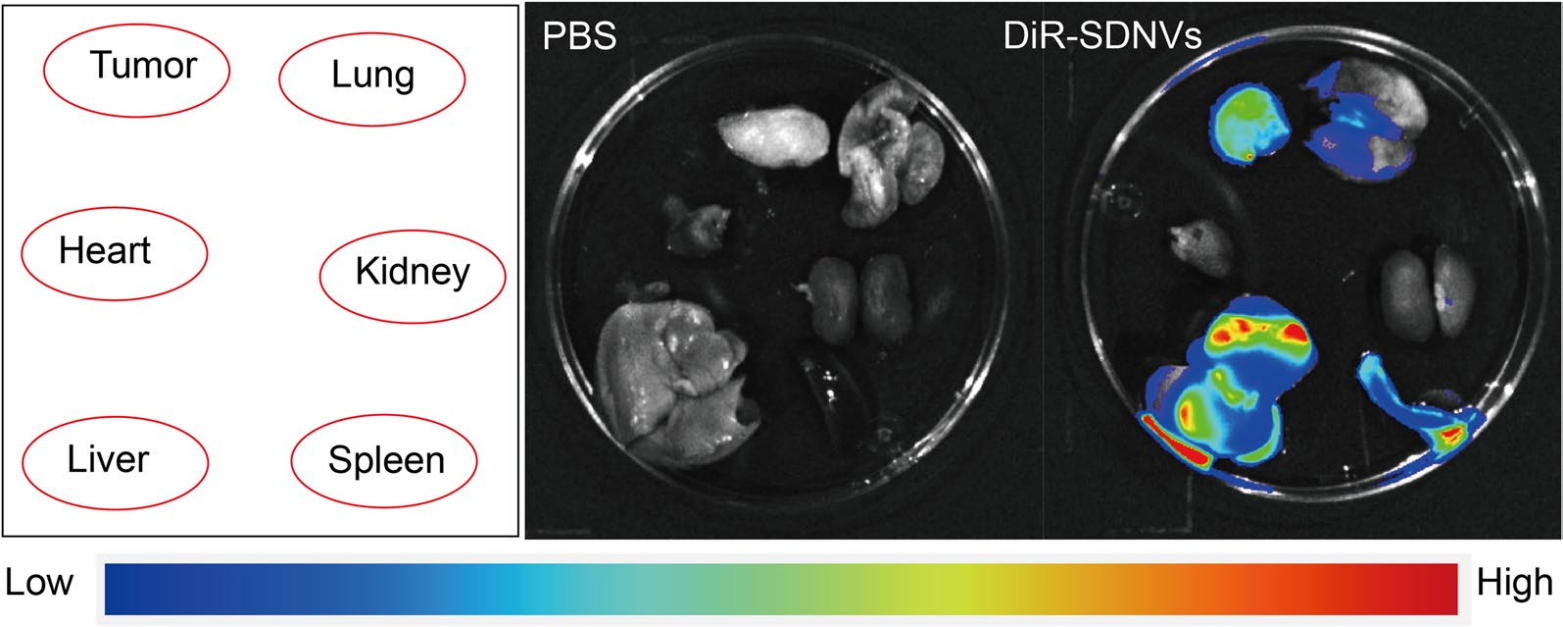
In vivo distribution of orally administered SDNVs. Representative ex vivo fluorescence images of major organs and tumor tissues collected 6 h after oral administration of DiR-labeled SDNVs (DiR-SDNVs) or PBS. The tissues included tumor, lung, heart, kidney, liver, and spleen, as indicated in the schematic diagram. The pseudocolor scale represents fluorescence intensity from low to high

To investigate the biodistribution of SDNVs following oral administration, DiR-labeled SDNVs (DiR-SDNVs) were gavaged into male nude mice. Relative to PBS-treated controls, the DiR-SDNV-treated mice displayed a pronounced increase in ex vivo fluorescence accumulation within hepatic, splenic, pulmonary, and tumor tissues (Fig. 2), indicating that SDNVs were not confined to the gastrointestinal tract after gavage but were distributed to multiple organs in vivo. At the same time, a clear accumulation signal was observed in tumors within this multi-organ distribution pattern, indicating that orally administered SDNVs can effectively reach the tumor site. These results indicate that orally administered SDNVs were absorbed from the gastrointestinal tract and subsequently distributed to multiple organs.

### SDNVs inhibit tumor growth in vivo

**Fig. 3:**
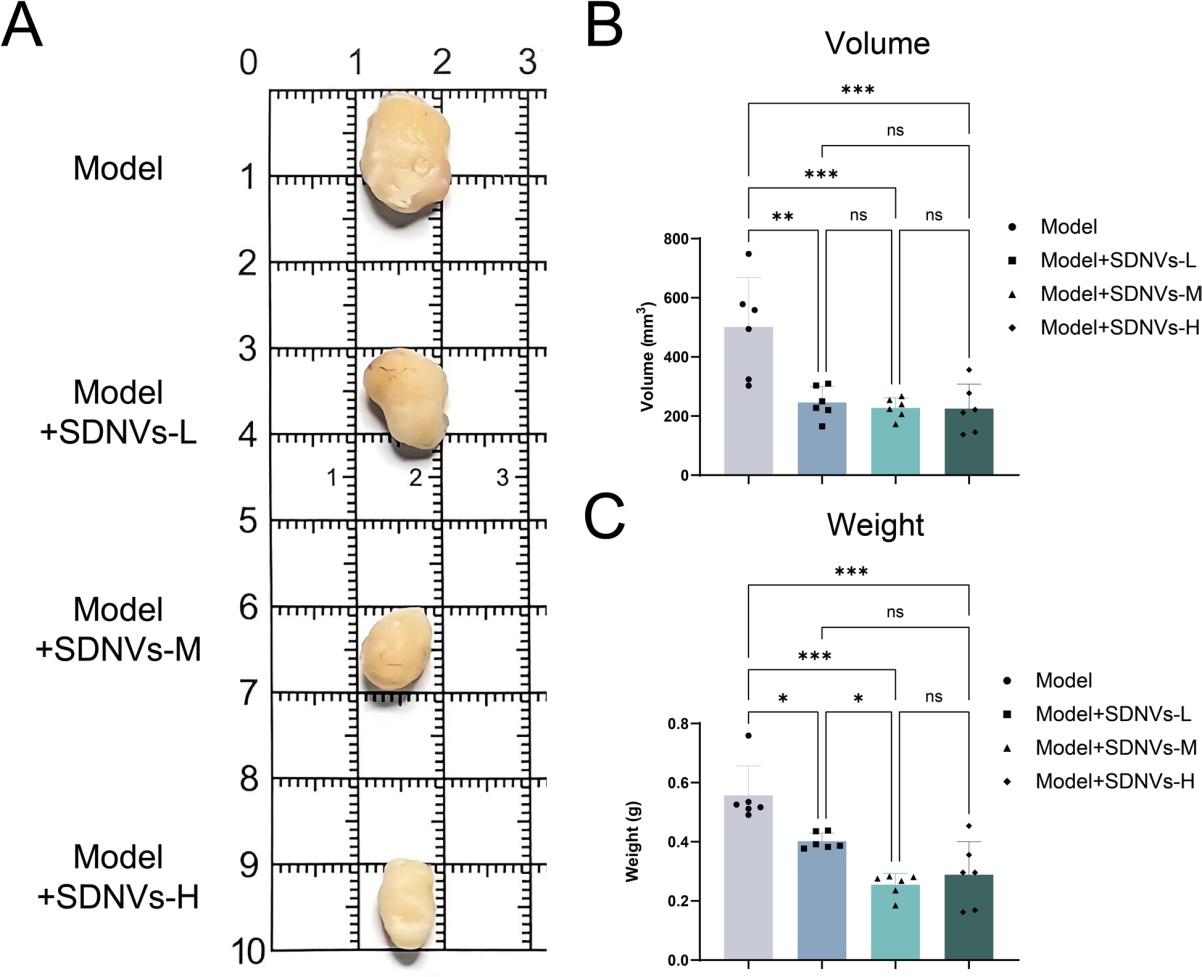
SDNVs inhibit tumor growth in a prostate cancer xenograft model. **A** Representative images of subcutaneous tumors from mice treated with PBS (Model) or SDNVs at low, medium, and high doses after 4 weeks of oral administration. **B** Tumor volume measurements over time showing significant growth inhibition in SDNV-treated groups (n = 6 per group). **C** Final tumor weights at sacrifice confirming reduced tumor burden in treated groups. Data are presented as mean ± SD; Statistical significance was denoted as follows: *p < 0.05, * *p < 0.01, ***p < 0.001, and ns for not significant

A xenograft mouse model of PCa was employed to determine whether SDNVs could suppress tumor development in vivo. Gross observation of subcutaneous tumors showed clear differences in tumor size between the model group and the SDNV-treated groups at low (L), medium (M), and high (H) doses (Fig. 3A), with visibly smaller tumors observed in all treatment groups than in the untreated model group. Measurement of tumor volume revealed that all three oral doses of SDNVs significantly reduced tumor growth relative to the model group. The extent of tumor inhibition was comparable among the low-, medium-, and high-dose groups, indicating that a robust antitumor effect was already achieved at the lowest tested dose and that further dose escalation primarily resulted in a plateau rather than a strictly linear dose–response (Fig. 3B). Consistently, tumor weight measurements showed a similar reduction pattern across all groups treated with SDNVs when referenced against model controls (Fig. 3C), further supporting the in vivo antitumor potential of SDNVs.

**Fig. 4:**
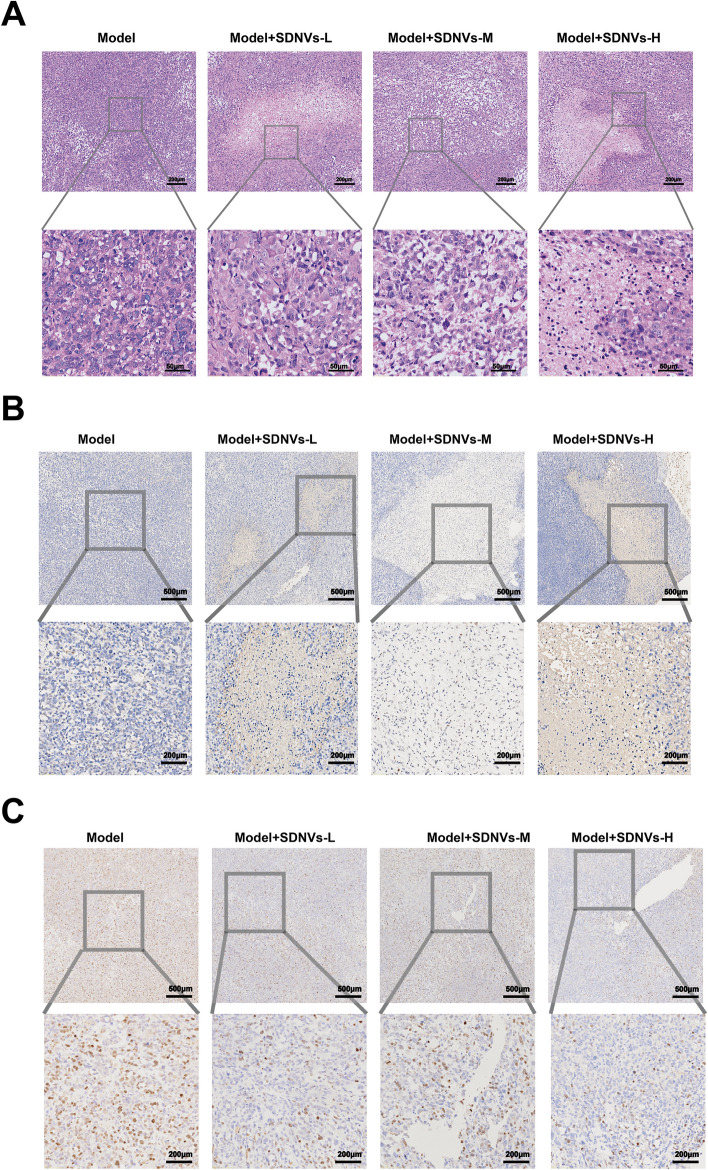
Histological and molecular evidence of SDNV-mediated tumor suppression. **A** H&E staining of tumor sections showing reduced cell density and nuclear atypia in SDNV-treated groups. **B** TUNEL staining indicating increased apoptotic cell death following SDNV treatment. **C** Ki-67 immunohistochemical staining demonstrating suppressed tumor cell proliferation in treated groups (n = 6 per group)

H&E staining showed that tumors in the model group exhibited high cellular density, disorganized architecture, and prominent nuclear atypia. In contrast, SDNV-treated tumors displayed decreased cell density, nuclear pyknosis, and more orderly arrangement, indicating a reduced malignancy, with these changes becoming more evident as the dose increased (Fig. 4A). The number of TUNEL-positive cells was clearly elevated after SDNV treatment, with the most pronounced changes observed in the high-dose group, suggesting a dose-associated pro-apoptotic effect of SDNVs on prostate cancer cells (Fig. 4B). In addition, Ki-67 immunostaining showed a high proportion of proliferating cells in control tumors, whereas SDNV treatment markedly reduced Ki-67 positivity (Fig. 4C). These results indicate that SDNVs effectively suppress prostate tumor growth in vivo by promoting apoptosis and reducing proliferation.

### Uptake and internalization of SDNVs by PC-3 cells

**Fig. 5:**
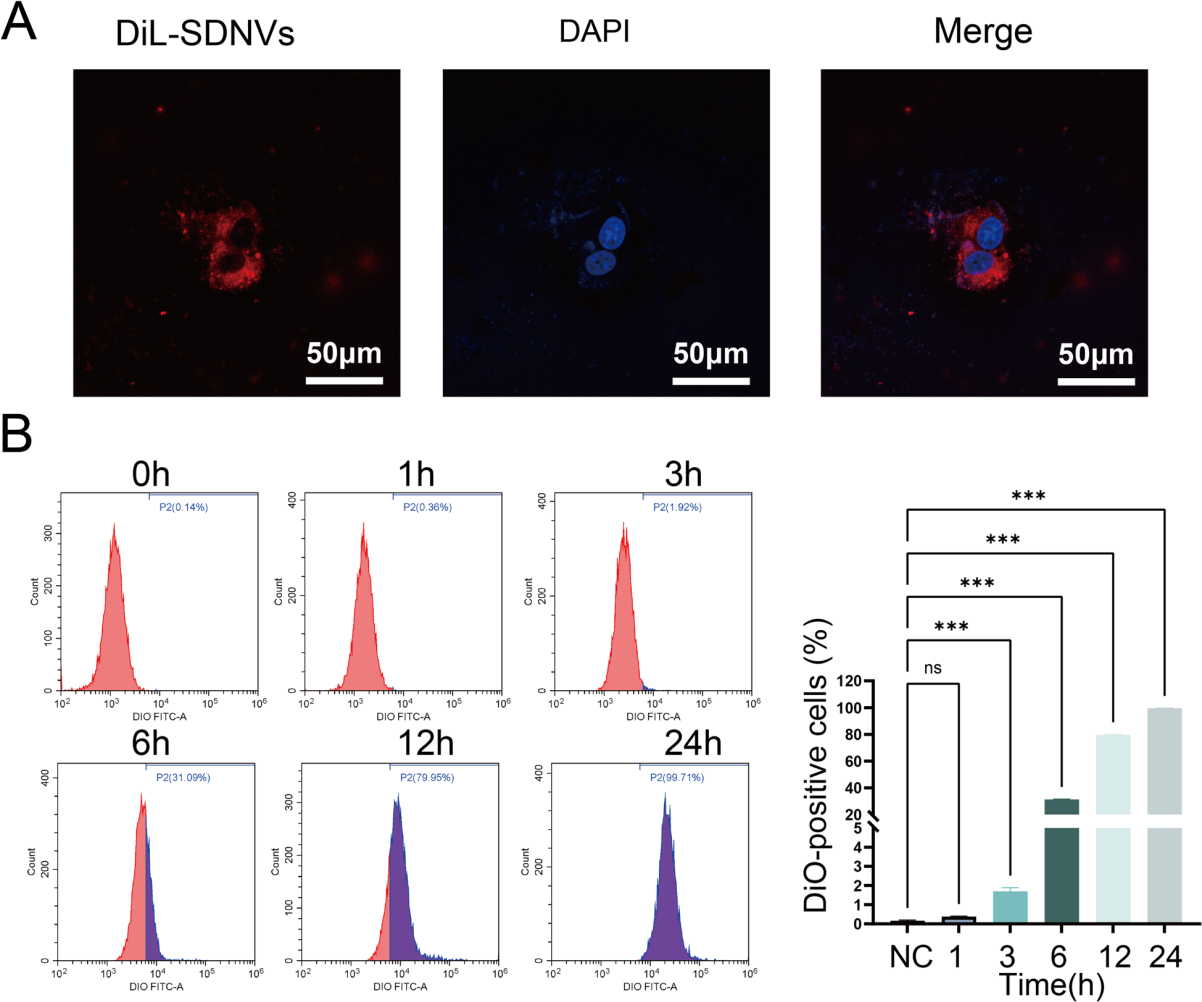
Cellular uptake and internalization of SDNVs by PC-3 cells. **A** Confocal laser scanning microscopy showing intracellular localization of DiI-labeled SDNVs after incubation with PC-3 cells. **B** Flow cytometry analysis of DiO-labeled SDNVs uptake in PC-3 cells at various time points, confirming efficient cellular internalization. Data are presented as mean ± SD; Statistical significance was denoted as follows: ***p < 0.001, and ns for not significant

We next investigated the uptake of SDNVs in PC-3 cells to better understand their cellular antitumor activity. SDNVs were labeled with the fluorescent dyes DiI and DiO to generate DiI-SDNVs and DiO-SDNVs, respectively. Confocal laser scanning microscopy revealed distinct red fluorescence within the cytoplasm of PC-3 cells after incubation with DiI-SDNVs, indicating efficient cellular internalization (Fig. 5A). To quantitatively assess the uptake kinetics, PC-3 cells were incubated with DiO-SDNVs and analyzed by flow cytometry at various time points. The results showed that cellular uptake began as early as 3 h and increased progressively over time, displaying a clear time-dependent pattern (Fig. 5B). By 24 h, nearly all PC-3 cells had internalized DiO-SDNVs.

### SDNVs exhibit antitumor activity in PC-3 cells in vitro

**Fig. 6:**
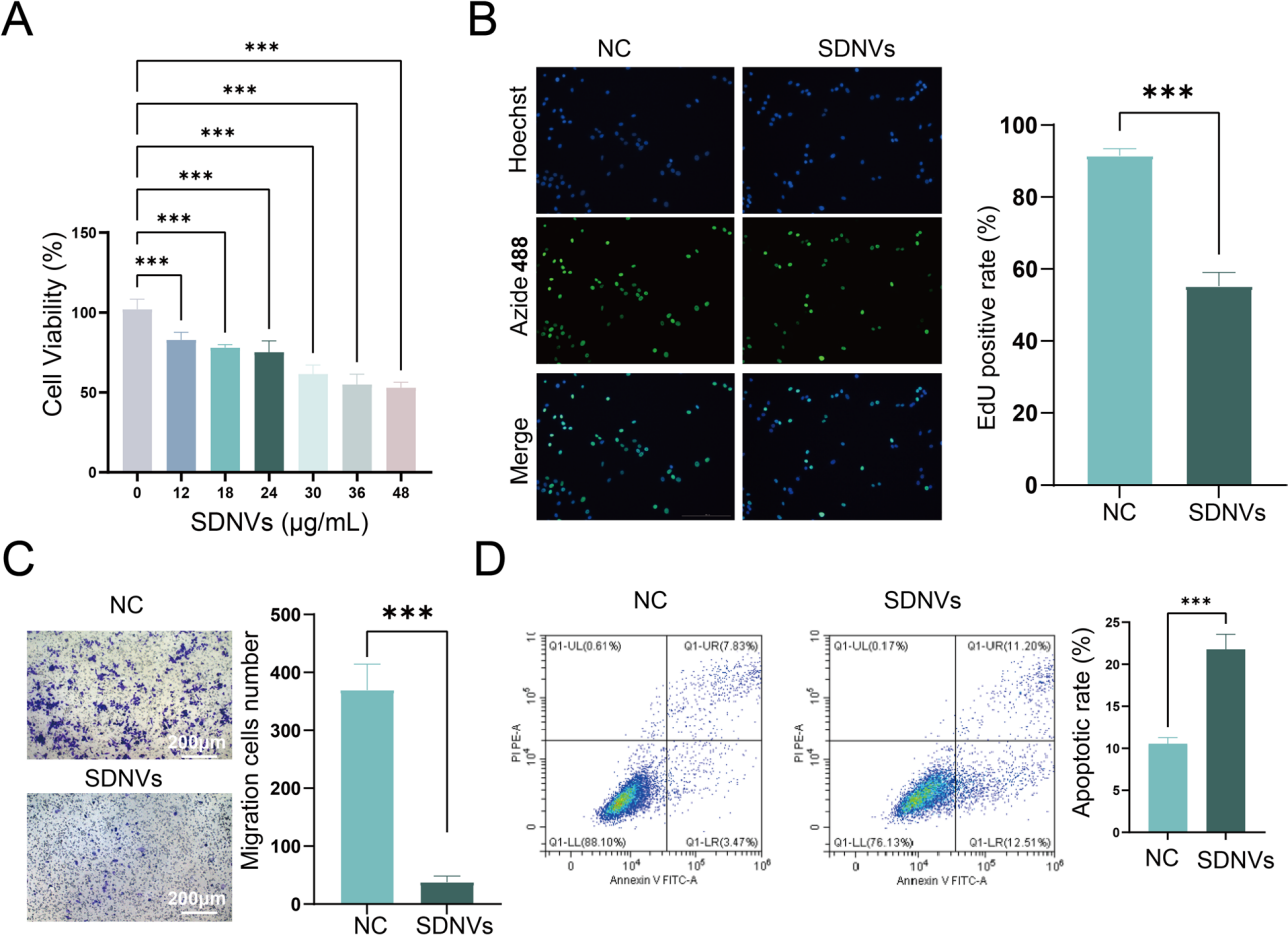
In vitro antitumor effects of SDNVs on PC-3 cells. **A** CCK-8 assay demonstrating dose-dependent reduction in PC-3 cell viability following SDNV treatment. **B** EdU assay showing decreased proliferation in SDNV-treated cells. **C** Transwell migration assay indicating reduced migratory capacity after SDNV intervention. **D** Flow cytometry analysis using Annexin V-FITC/PI staining revealing increased apoptosis in SDNV-treated PC-3 cells. Data are presented as mean ± SD; Statistical significance was denoted as follows: ***p < 0.001, and ns for not significant (n = 3)

Although SDNVs exhibited clear tumor-inhibitory effects in vivo, we further conducted in vitro experiments to determine whether these effects were due to a direct action on cancer cells and to clarify the specific cellular processes involved. To define the functional basis of SDNV’s tumor suppression, we examined the viability, proliferation, migration, and apoptosis of PC-3 cells. CCK-8 results indicated that higher concentrations of SDNVs were associated with lower viability of PC-3 cells, reaching approximately 60% at 36 μg/mL (Fig. 6A). EdU incorporation analysis further supported the antiproliferative effect of SDNVs, showing a marked reduction in DNA synthesis in treated PC-3 cells relative to the control group (Fig. 6B). In addition, transwell migration assays revealed a significant inhibition of cell motility following SDNV treatment, suggesting a suppressive effect on invasive potential (Fig. 6C). Relative to controls, SDNV exposure markedly increased the proportion of apoptotic PC-3 cells (Fig. 6D), suggesting that SDNVs trigger programmed cell death. These in vitro findings complement the in vivo results and demonstrate that SDNVs directly inhibit prostate cancer cell growth through a combination of antiproliferative, antimigratory, and pro-apoptotic effects.

### Transcriptomic analysis suggests activation of p53/p21-associated senescence pathways

**Fig. 7:**
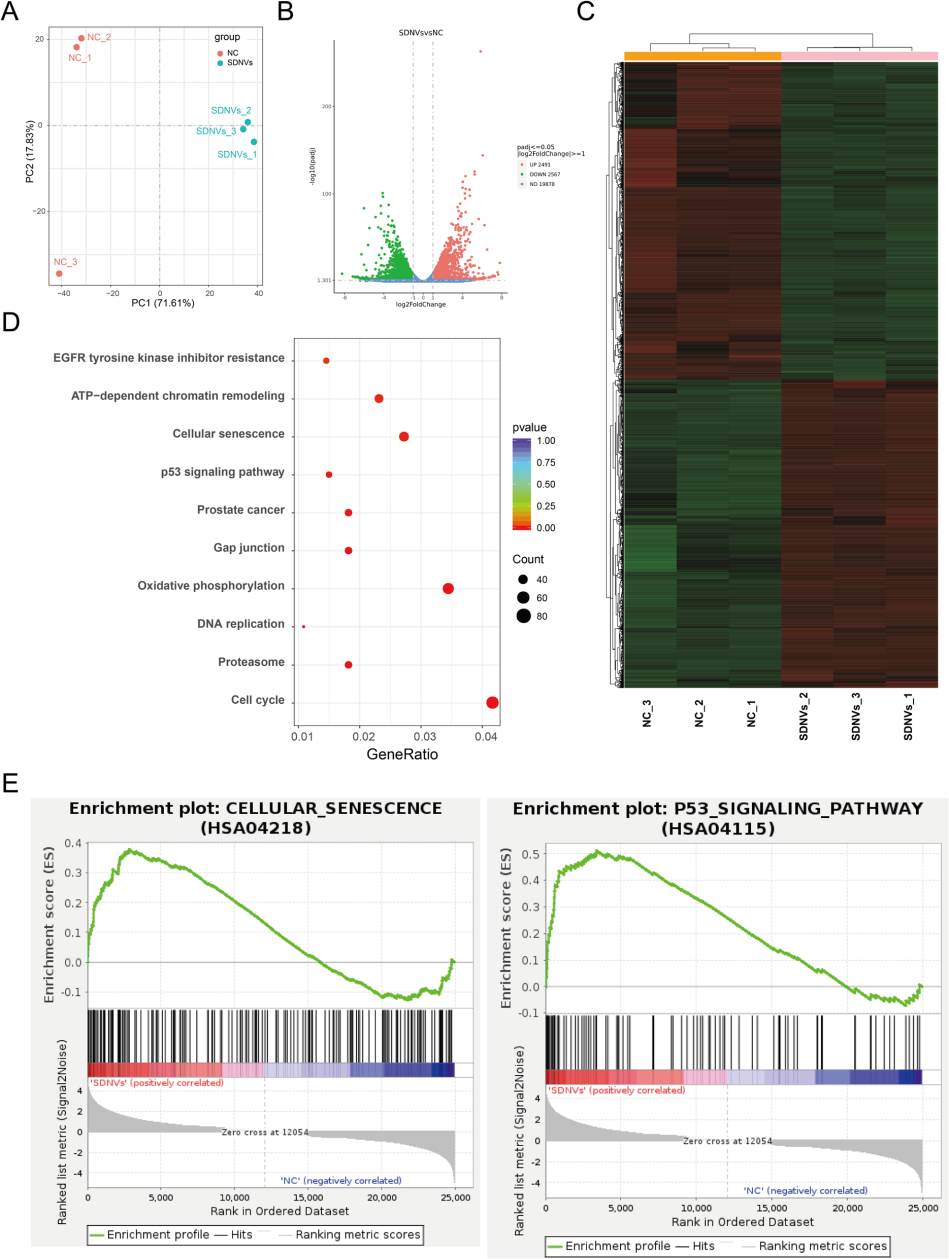
Transcriptomic profiling reveals SDNV-induced activation of senescence and p53 signaling in PC-3 cells. **A** Principal component analysis (PCA) showing distinct clustering of SDNV-treated samples compared to controls. **B** Volcano plot illustrating 2491 upregulated and 2567 downregulated genes in SDNV-treated cells (adjusted P < 0.05). **C** Heatmap of differentially expressed genes (DEGs) demonstrating consistent transcriptional alterations across replicates. **D** KEGG enrichment analysis showing significant involvement of DEGs in the p53 signaling pathway, cellular senescence, and prostate cancer-related pathways. **E** Gene set enrichment analysis (GSEA) confirming activation of cellular senescence and the p53 pathway following SDNV treatment

To explore the molecular mechanisms of SDNV-mediated antitumor activity, transcriptomic analysis was conducted in SDNV-treated PC-3 cells. Principal component analysis (PCA) showed that SDNV-treated samples clustered closely together, indicating good reproducibility and a distinct transcriptional profile compared to controls (Fig. 7A). Differential expression analysis identified 5058 DEGs, with 2491 transcripts being upregulated and 2567 being downregulated (Fig. 7B). A volcano plot highlighted significant gene expression shifts, while unsupervised hierarchical clustering revealed clear segregation between SDNV-treated and control groups, reflecting widespread transcriptional reprogramming (Fig. 7C). Pathway analysis revealed prominent enrichment of the identified genes in senescence-associated processes, p53-related signaling, and prostate cancer-related pathways (Fig. 7D). Consistently, GSEA further supported the activation of both the cellular senescence program and the p53 pathway in SDNV-treated cells.

### SDNVs promote a senescence-like phenotype in PC-3 cells through p53/p21 signaling

**Fig. 8:**
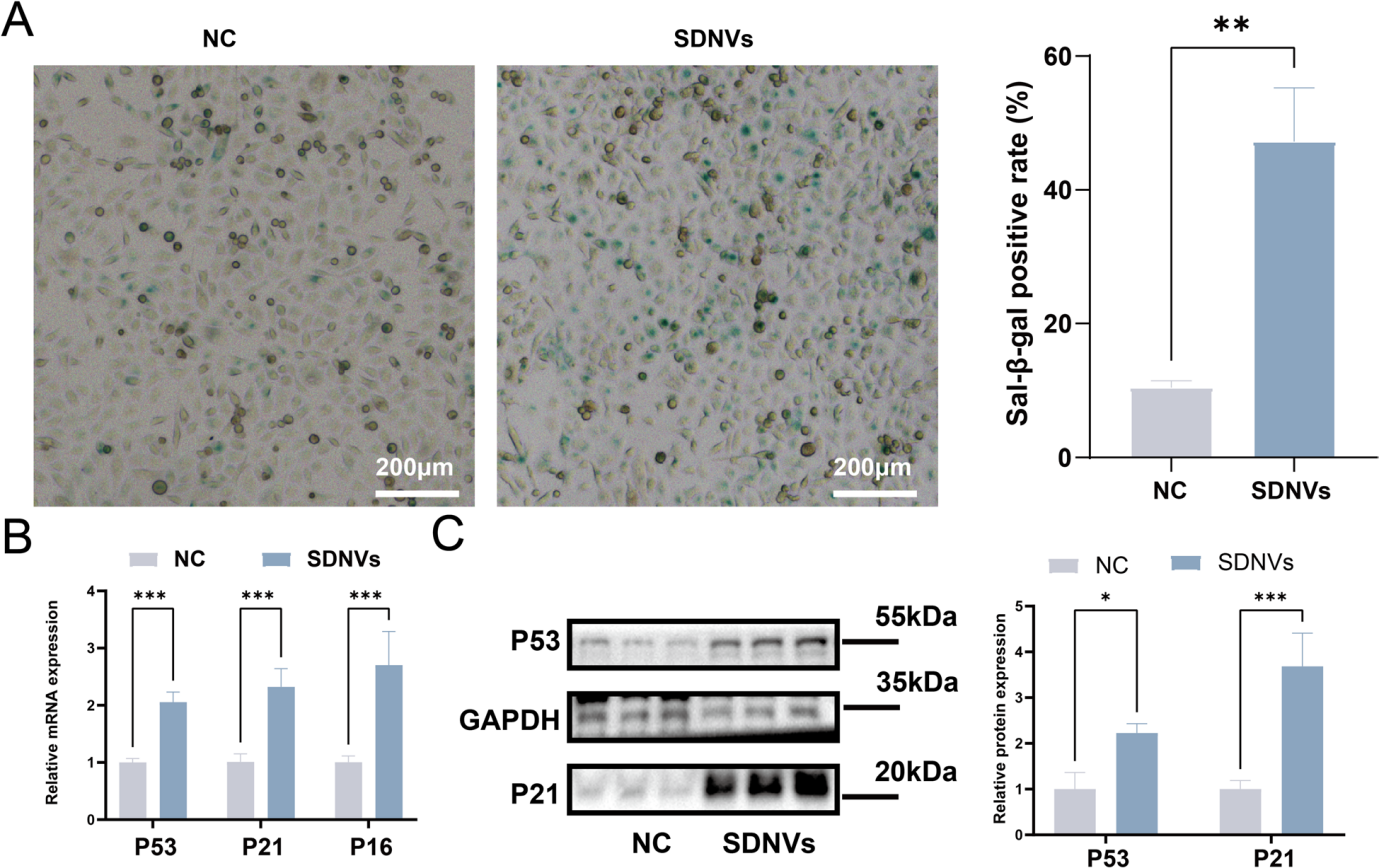
SDNVs induce cellular senescence in PC-3 cells via activation of the p53/p21 pathway. **A** SA-β-gal staining of PC-3 cells after SDNV treatment showing increased proportion of blue-stained senescent cells compared to controls. **B** Quantitative real-time PCR analysis showing upregulation of P53, P21, and P16 mRNA levels in the SDNV-treated group. **C** Western blot analysis confirming elevated expression of P53 and P21 proteins in response to SDNV treatment, with GAPDH as a loading control. Data are presented as mean ± SD; Statistical significance was denoted as follows: *p < 0.05, * *p < 0.01, ***p < 0.001, and ns for not significant (n = 3)

Based on transcriptomic sequencing and pathway enrichment analysis, SDNVs were hypothesized to exert antitumor effects through the induction of cellular senescence. To validate this, a SA-β-gal staining assay was conducted. Compared with the NC group, SDNV-treated PC-3 cells exhibited markedly increased SA-β-gal staining and more positive cells, indicating the induction of a senescent phenotype (Fig. 8A). To further assess pathway activation, qPCR was carried out for P53 and its downstream senescence-related targets P21 and P16. SDNV treatment significantly upregulated the transcription of all three genes (Fig. 8B), suggesting an important role for P53 signaling in SDNV-induced senescence. Consistently, P53 and P21 protein abundance were also increased in SDNV-treated cells relative to the NC group (Fig. 8C), further supporting activation of the P53 pathway at the protein level.

### PFT-α inhibits SDNV-induced senescence via p53 pathway suppression

**Fig. 9:**
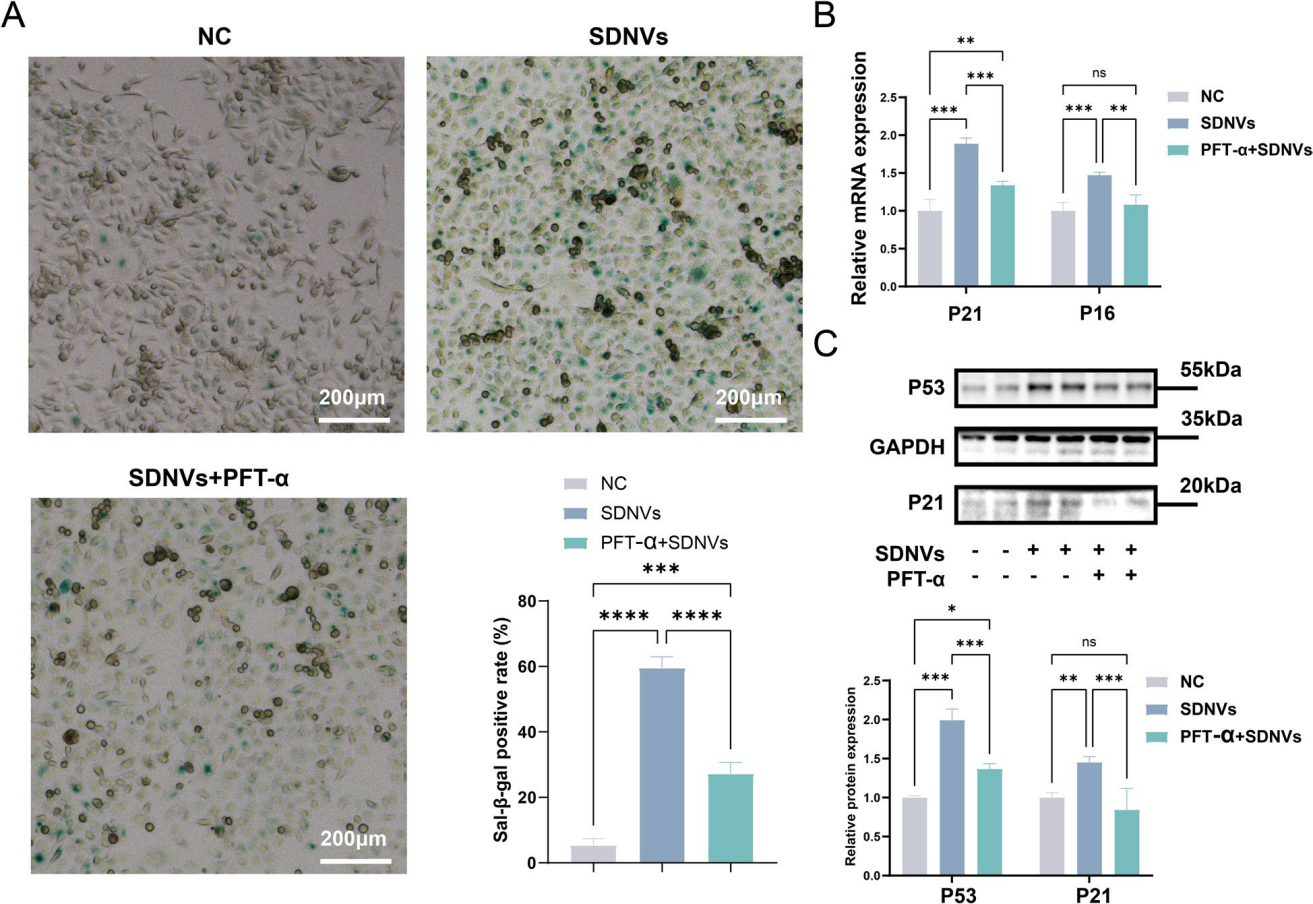
PFT-α attenuates SDNV-induced cellular senescence and suppresses activation of the p53 pathway in PC-3 cells. **A** SA-β-gal staining showing that PFT-α treatment reduced the proportion of senescent cells induced by SDNVs in PC-3 cells. **B** qPCR analysis indicating that PFT-α downregulated SDNV-induced mRNA expression of p53 pathway-related genes (P21 and P16). **C** Western blot analysis demonstrating that PFT-α inhibited SDNV-induced upregulation of p53 and p21 protein expression. Data are presented as mean ± SD from three independent experiments. Statistical significance was denoted as follows: *p < 0.05, * *p < 0.01, ***p < 0.001, and ns for not significant (n = 3)

The role of p53 signaling in the SDNV-driven senescence-like phenotype was further examined by co-incubating PC-3 cells with SDNVs and PFT-α (20 μM), a reversible inhibitor of p53 transcriptional activity, and then performing SA-β-gal staining. Compared to the SDNV-only group, the SDNVs + PFT-α group exhibited a marked reduction in blue-stained senescent cells under light microscopy, indicating that PFT-α effectively attenuated the pro-senescent effect of SDNVs (Fig. 9A). To investigate whether this effect was mediated through suppression of P53 downstream targets, we quantified the expression of key pathway components. qPCR analysis revealed lower P21 and P16 mRNA levels after co-treatment with SDNVs and PFT-α than after SDNV treatment alone (Fig. 9B). Similarly, Western blot further showed decreased protein expression of P53 and P21 following PFT-α treatment (Fig. 9C).

### Safety evaluation of SDNVs

**Fig. 10:**
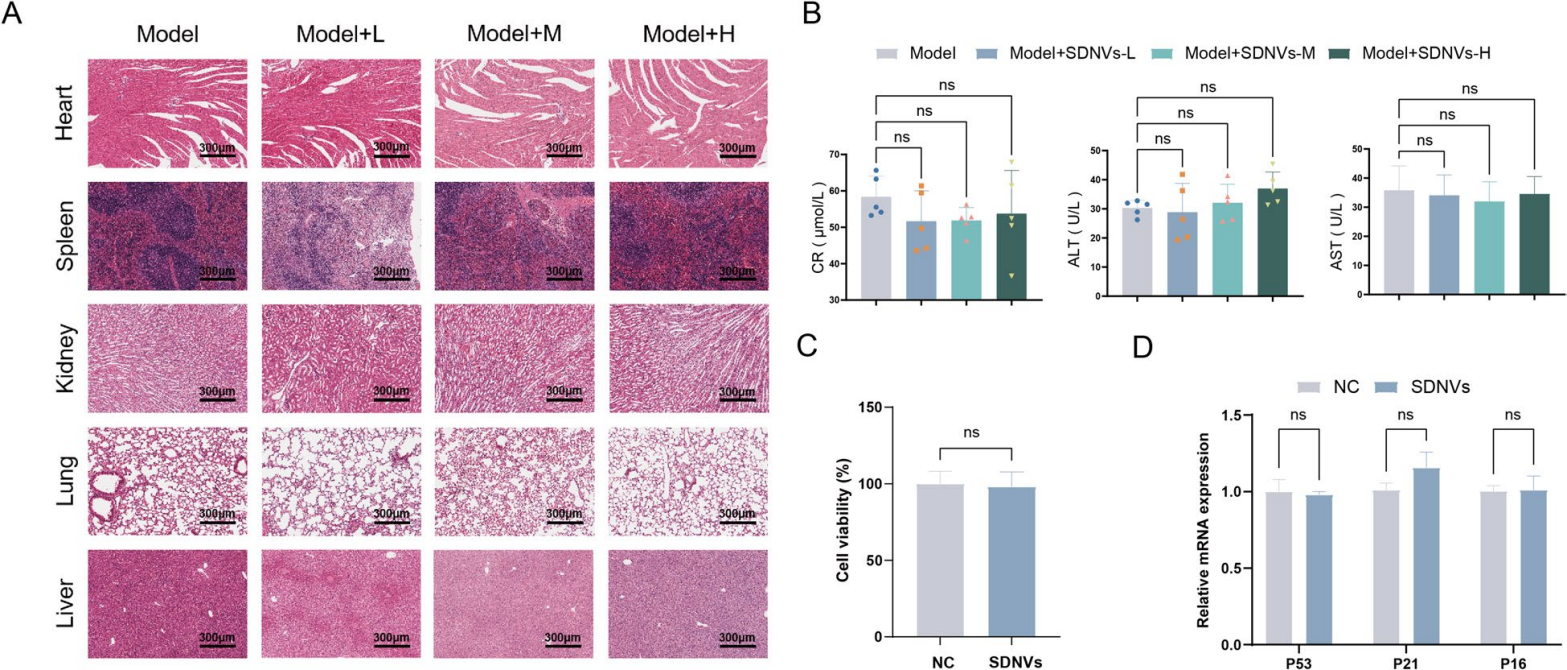
In vivo and in vitro safety evaluation of SDNVs. **A** Histological examination (H&E staining) of major organs (heart, liver, spleen, lung, and kidney) from mice treated with various doses of SDNVs, showing no observable pathological changes. **B** Serum biochemical analysis of liver and kidney function markers (ALT, AST, and CRE) indicating no significant toxicity following SDNV administration. **C** CCK-8 assay evaluating the effect of SDNVs on viability of RWPE-1 normal prostate epithelial cells. **D** qPCR analysis showing that SDNVs did not significantly alter the expression of senescence-associated genes (P53, P21, and P16) in RWPE-1 cells. Data are presented as mean ± SD from three independent experiments. ns: *p* > 0.05

The biosafety of SDNVs was examined using both animal and cell-based assays. For the in vivo study, mice received oral administration of SDNVs at varying doses. H&E-stained sections of cardiac, pulmonary, hepatic, splenic, and renal tissues showed no evident structural injury or pathological lesions in any treatment group, indicating that SDNVs did not induce histological toxicity (Fig. 10A). Furthermore, serum biochemical indices of liver and kidney injury markers such as CRE, ALT, and AST, stayed within normal limits after SDNV administration, showing no statistically significant differences from the control group (Fig. 10B). These results verify that SDNVs do not cause hepatotoxicity or nephrotoxicity in vivo. In vitro safety was evaluated using RWPE-1 normal human prostate epithelial cells. CCK-8 assay indicated that SDNVs did not significantly impact cell viability (Fig. 10C). Additionally, no significant changes were detected by qPCR in the transcript levels of the aging-related markers P53, P21, and P16 after SDNV treatment (Fig. 10D). Taken together with the multi-organ biodistribution in Fig. 2, these findings indicate good tolerability of SDNVs at the tested doses. There is no apparent histological or biochemical harm caused by SDNVs in major organs or normal prostate epithelial cells. Moreover, these safety data support their potential as a safe nanotherapeutic platform for further application.

## Discussion

This study provides the first evidence that SDNVs exert selective antitumor effects against PCa. Orally administered SDNVs significantly inhibited tumor growth and induced cellular senescence in PC-3 cells, while showing minimal effects on normal prostate epithelial cells. Further analysis indicated that the p53/p21 pathway activation is crucial in mediating these biological effects. These findings highlight SDNVs as a safe and biocompatible nanoplatform with promising therapeutic potential in PCa.

PDNVs are increasingly recognized as a promising class of bioactive nanomaterials for disease intervention. Their favorable biocompatibility, minimal immune response, and capacity to encapsulate and transport diverse functional cargos make them attractive candidates for therapeutic applications. Accumulating evidence has shown that PDNVs derived from edible or medicinal plants can inhibit tumor progression by apoptosis, suppressing epithelial-mesenchymal transition, and modulating the immune response. Aloe vera-derived nanovesicles inhibit pancreatic cancer progression by inducing pyroptosis through the ROS-GSDMD/E signaling pathway (Shen et al. 2025). In addition, *Citrus limon*-derived nanovesicles showed antitumor activity across several malignant cell models and restrained CML progression in vivo after intratumoral or intraperitoneal administration. Notably, these vesicles were detected at the tumor site in vivo and were associated with enhanced TRAIL-mediated apoptotic signaling (Raimondo et al. 2015). Despite these advances, current evidence regarding PDNV use against prostate tumors is still scarce, with no available reports on cellular senescence as a therapeutic mechanism. Most existing approaches emphasize cytotoxicity-based interventions, while strategies that selectively activate tumor suppressive senescence pathways remain underdeveloped. These data support a previously unreported function of Solanum nigrum L.-derived nanovesicles (SDNVs) in limiting prostate cancer progression through p53/p21-driven senescence, with minimal impact on normal cells. This non-cytotoxic mode of action represents a novel perspective in PDNV-based therapy, potentially reducing adverse effects associated with traditional chemotherapeutics.

Rather than simple cell-cycle arrest, cellular senescence represents a persistent stress-response state induced by genotoxic, oxidative, or oncogenic cues. Its hallmark changes include checkpoint engagement involving p16/Rb and p53/p21, increased SA-β-gal activity, chromatin remodeling, and secretion of senescence-associated factors (Hernandez-Segura et al. 2018; Gorgoulis et al. 2019). In cancer biology, cellular senescence plays dual roles: initially restricting tumor progression through growth arrest and immune-mediated clearance, but potentially driving tumorigenesis through prolonged SASP-induced inflammation and immune evasion (Schosserer et al. 2017). Recent studies have identified PDNVs as novel regulators of senescence in various biological systems (Karabay et al.^2025^). Notably, *Semen Sinapis alba*-derived nanovesicles have been shown to mitigate endothelial senescence and hypertensive arterial remodeling in spontaneously hypertensive rats by delivering miR393a to suppress CD38 expression and downregulate the p53/p21 axis (Lin et al. 2025). Similarly, *Ecklonia cava*-derived nanovesicles attenuated redox imbalance and senescence-associated changes in keratinocytes and aged mice, accompanied by enhanced HSP70 expression and reduced TNF-α, MAPK, and NF-κB signaling (Batsukh et al. 2024). Nanovesicles derived from *Phellinus linteus* were likewise found to mitigate photoaging caused by ultraviolet exposure (Jingxia et al. 2022). In our previous research, nanovesicles derived from *Dendrobium* were found to enhance wound healing, likely by mitigating cellular senescence (Tu et al. 2024, 2025).

In this study, transcriptomic analysis highlighted significant activation of senescence-related genes and the p53 signaling pathway in SDNV-treated PC-3 cells. Functional assays verified a senescence-like phenotype, characterized by enhanced SA-β-gal positivity, morphological remodeling, and increased expression of p16, p21, and p53. The tumor-suppressive role of p53 is well documented, with loss of its function being closely linked to aggressive behavior, recurrence, and poor prognosis in PCa (Tornesello 2025). Both p21 and p16 serve as key cyclin-dependent kinase inhibitors that mediate G1-phase arrest and enforce stable senescence (Rayess et al. 2012; Rane and Minden 2019). Importantly, the senescence-associated phenotype was markedly attenuated after p53 blockade with PFT-α, supporting a central role for p53/p21 signaling in the action of SDNVs. No previous study has directly shown that PDNVs can induce therapy-related senescence in cancer cells, offering a non-cytotoxic mechanism of action and broadening the potential application of PDNVs in oncological treatment strategies.

Although the present results support an antitumor effect of SDNVs associated with senescence induction, several limitations should be acknowledged. Firstly, the exact bioactive components within SDNVs responsible for triggering senescence remain unidentified. Metabolomic analyses indicated unique chemical profiles distinct from traditional aqueous extracts, yet further studies are necessary to pinpoint active metabolites or functional RNAs contributing to the observed effects. Secondly, our animal model utilized subcutaneous xenografts, which offer limited representation of the stromal environment, immune landscape, and metastatic potential characteristic of human PCa. Future studies incorporating orthotopic or genetically engineered mouse models would provide more clinically relevant insights. Thirdly, our biodistribution and safety data indicate that orally administered SDNVs reach multiple organs and are well tolerated at the tested doses. However, we did not evaluate more subtle or long-term off-target effects in non-tumor tissues, which will require dedicated toxicological studies in future work. Finally, more detailed studies are required to characterize the immune-related consequences of SDNV-induced tumor senescence, with particular attention to SASP-associated signaling and its impact on disease progression in immunocompetent hosts. Despite the aforementioned limitations, our findings highlight SDNVs as a promising candidate for clinical translation due to their selective antitumor activity, oral bioavailability, and excellent biosafety profile. Unlike conventional cytotoxic therapies, SDNV-mediated induction of cellular senescence provides a non-lethal strategy that could reduce the adverse effects commonly associated with chemotherapy and radiotherapy.

## Conclusion

In summary, our study shows that SDNVs selectively inhibit PCa progression by inducing cellular senescence through the p53/p21 signaling pathway. Oral administration of SDNVs effectively suppressed tumor growth in vivo and exhibited excellent safety and biocompatibility, supporting their potential as a novel, non-cytotoxic therapeutic strategy.

## Supplementary Information

Supplementary Material 1.

## Data Availability

The data supporting the findings of this study are included in the main text and the Supplementary Information. Further information is available from the corresponding authors upon reasonable request.
