# Supplementary material for: Solanum nigrum L.-derived nanovesicles as novel nanotherapeutics suppressing prostate cancer progression via senescence-based antitumor activity
Source: Bioresour Bioprocess. 2026 Apr 27;13(1):56. doi: 10.1186/s40643-026-01055-y (PMC13121694; doi:10.1186/s40643-026-01055-y)
Supplement: Supplementary file 1 — Supplementary Material 1. [file 40643_2026_1055_MOESM1_ESM.doc]

**1. Supplementary table**

**Table S1 Primers for quantitative RT‒PCR analysis**

| Name | Sequence (5′→3′) |
| --- | --- |
| *hsa-ATCB* | F: CATGTACGTTGCTATCCAGGC |
|  | R: CTCCTTAATGTCACGCACGAT |
| *hsa-TP53* | F: :GAGGTTGGCTCTGACTGTACC |
|  | R: TCCGTCCCAGTAGATTACCAC |
| *hsa-P21* | F: GGAACTTCGACTTTGTCACCG |
|  | R: GCACAAGGGTACAAGACAGTG |
| *hsa-P16* | F: GGTGCGCAGGTTCTTGGTGAC |
|  | R: CTAGACGCTGGCTCCTCAGTA |
